# Supporting Young Carers in Early Childhood: Mapping Power, Threat, Meaning, and Strengths: A PTMF-Informed Qualitative Study

**DOI:** 10.3390/healthcare14020213

**Published:** 2026-01-14

**Authors:** Carly Ellicott, Ali Bidaran, Felicity Dewsbery, Alyson Norman, Helen Lloyd

**Affiliations:** 1 School of Psychology, Faculty of Health, University of Plymouth, Plymouth PL4 8AA, UK; 2 Pen Green Centre for Children and their Families, Corby NN171BJ, UK

**Keywords:** young carers, early childhood, infant mental health, mental health, well-being, safeguarding, whole family support

## Abstract

**Background/Objectives:** This qualitative study examines strengths and strains faced by professionals working with young carers throughout the United Kingdom (UK) in the context of society’s youngest carers; young carers in early childhood (YCEC) (0–8 years). **Methods:** The Power Threat Meaning Framework (PTMF) was utilised to map key findings of three focus groups. This conceptual lens offers a narrative-based understanding of ways in which power operates in society. Increasingly applied to explore experiences of individuals, communities, and groups, the PTMF proposes that concepts of distress are founded in broader contexts of injustice and social inequalities. Twenty-four participants were recruited from throughout the UK via the Carers Trust Young Carers Alliance. **Results:** Findings highlight the strength of legal, ideological, and economic power shaping societal beliefs and policy concerning YCEC. This informs constructs of perceived social norms regarding who young carers are most likely to be, and where they may be found. This power threatens the health and well-being of YCEC, impacting the ability of professionals to provide optimal support. Inappropriate policy formed from these assumptions disempowers those providing services to young carers at the frontline of service delivery. Professionals and adults with living experience of caring in their early childhoods reflect upon silent tensions that exist within society, suggesting that YCEC remain the ‘elephant in the room’. **Conclusions:** We make recommendations to review the efficacy of statutory mandates concerning the needs assessment of young carers in England, and to align policy concerning early childhood and young carers to embed young carers’ rights consistently, starting in early childhood.

## 1. Introduction

In the United Kingdom (UK), there is a lack of research centred on young carers in early childhood (YCEC) (0–8 years), very young children providing or intending to provide care to a family member. This is also mirrored by the very few opportunities that have been made to empower the voice of YCEC through policy, practice, or representation in broader young carer discourse [1,2,3]. Where representation is present, it is projected through a safeguarding lens where young children are impacted by family members requiring care to manage illness, disability, addiction, and/or mental health needs. The intention is to protect young children through robust legislation to prioritise their wellbeing [4,5,6,7,8]. This lens shapes a discourse that caring activity for YCEC is wholly inappropriate, particularly when children are still in their early years (0–5 years) [9]. Yet, young carers as young as three and four years old in the UK shoulder a significant weight of responsibility, which is predominantly under-identified for up to ten years [10,11]. In such cases, a safeguarding lens fails to expand a safety net of risk prevention to YCEC. This is evident within serious case reviews, which repeatedly highlight failings to implement whole family, whole system working practices along a continuum of effective support. Such failures can lead to irrevocable consequences, including death by suicide [12,13].

Existing research tells us that identification of young carers is often delayed, and the needs of young carers become more apparent when a family has reached a state of crisis [14]. Where known vulnerabilities of safeguarding are acknowledged, a child’s caring role can often remain overlooked [12]. Furthermore, services commissioned throughout the UK to provide needs assessments of young carers, as well as services of support and intervention, are not consistently well-resourced or trained to meet the specific developmental needs of society’s youngest carers [1,2,3].

In England, every local authority must comply with the statutory instrument Young Carers (Needs Assessment) Regulations 2015. This stipulates that local authorities *“must do what is possible to reach out to young carers and their families to prevent young carers from remaining hidden in local communities”.* [15] (p. 12). The strength of legislation influences the ranking of the UK as delivering a level 2 response to young carer awareness internationally [16]. Leu et al. [16] (p. 623) suggest that at this level, the UK offers “*widespread awareness and recognition of young carers amongst public, policy makers and professionals… and multiple dedicated services and interventions”* are available. However, evidence suggests that early intervention strategies do not robustly prevent serious harm or mitigate the risk associated with young caregiving sufficiently [13,17]. In the serious case review (SCR) conducted on behalf of Millie [17] (youngest of four siblings, living in the sole care of their father, identified in the review as a single carer facing difficulty prioritising the needs of his children over his own mental health), records indicate her caring responsibilities were acknowledged, yet misunderstood by professionals.

“Millie told School Nurse Services about her anxiety and that she felt stressed, was often lonely, sometimes angry, and had other matters that were worrying her. Further, this is the first occasion that she indicated that she looked after her dad by giving him tablets and water. Plus, she was worried about the failing health of her grandmother” [17] (p. 9).

Echoing experiences reported in Ellicott et al.’s [3] YCEC case study paper, the SCR acknowledges that engagement with professionals did not equate to recognition of very young children as carers. Nor was their vulnerability to take on increasing caring responsibility over time. Millie’s SCR does not specify if Millie received a young carer’s needs assessment in the years that led to her death. Notably absent is the term young carer, negating her complex needs and experiences within the review itself. By the age of 11 years, Millie had died by suicide [17].

Her story does not rest in isolation. A qualitative US study conducted by Ruch et al. [18] concluded that death by suicide amongst children aged between five and eleven years is the eighth leading cause of death amongst children in America. In England, suicide is deemed one of the leading causes of death in children and young people. The Office for National Statistics (ONS), England, recorded 345 deaths by suicide between 2011 and 2022 of children aged between four and nine years of age [19]. Common predisposing factors identified in reports cite multiple contributory factors, including exposure to mental health issues, early trauma, family environment, and bereavement. There is also recognition that factors are cumulative over time [18,19,20,21,22,23]. Further research and analysis are required to better understand the deeply sensitive and complex circumstances that lead very young children to suicide in the context of young caregiving. This is imperative in relation to broader social determinants of health, of which demand for healthcare services and early intervention play an integral role [24,25].

The Young Carer prevalence data in itself is thought to underestimate the number of children and young people with known caring responsibilities in the UK, and indeed internationally. Despite known vulnerabilities (low socioeconomic status, family environment/structure, living in areas of social deprivation), the prevalence of young carers aged between 5 and 17 years has risen in England since the COVID-19 pandemic [26]. Letelier et al. [26] suggest that study samples are not always representative of the range of factors associated with caregiving, and significant knowledge gaps remain. Furthermore, in England, the Education Committee Children’s Social Care report recognised a rise in cases of neglect, concluding the Department for Education has yet to develop a sufficient strategy to tackle these issues [27].

Recent changes to regulatory inspections in England have made explicit reference to young carers, placing greater accountability on structural containers (schools and social care providers) to identify and support young carers [28,29,30]. Yet, such amendments have not adopted advocacy of young carer legal rights consistently across all remits. Specifically notable is a failure to draw upon the skills and experience of early support within the Early Childhood Care and Education sector [31]. Brimblecombe et al. [32] suggest that the lack of resources remains a common barrier, perpetuating the unmet needs of young carers. Harnessing opportunities to develop existing modes of whole-family assessment through greater engagement and collaboration with the early childhood care and education sector (ECCE) could help to embed the rights mandated to all young carers more equitably. This would present an opportunity to develop a stronger commitment to the provision of a level 1 response to young carers nationally. Described as broadening responsiveness to achieve “*extensive awareness at all levels of government and society of the experiences and needs of young carers*”, this must apply to all ages [16] (p. 623). In doing so, UK practice would pave the way for other countries to develop preventive strategies to reduce risks associated with very young caregiving.

### 1.1. Why Does It Matter?

Broadening young carer narrative discourse relies on creating opportunities for inter-professional learning to take place. This can be described as a process by which two or more different professionals learn together to improve collaborative practice and person-centred outcomes [33]. Rose and Norwich [34] conceptualise inter-professional and multiagency working as framed in the context of the micro-level individual experience within a group. The individual lens influences group dynamics, which in turn impacts the collective motivation and effectiveness of a group’s ability to achieve a common goal. In this case, to develop stronger preventative strategies and mitigation of risk associated with caring responsibilities amongst YCEC. In context, a previous small-scale study carried out by the lead author indicated that professionals involved in the provision of young carer support are not always trained or experienced in supporting the developmental needs of young children. Where local authorities commission age-specific support for young carers, YCEC are often excluded [1]. Consequently, the young carer policy lacks insight and expertise from the field of early childhood studies [2].

Draper et al. [35] (p. 2094) discuss key interventions required during early childhood globally for optimal development during what has been termed the ‘next 1000 days’; describing the period in a child’s life extending from the first critical 1000 days (conception to two years of age) and the next period of two to five years. The paper highlights a need for policy environments that prioritise both ‘investment in ECCE systems and foster demand for services.’ Developing strategies to better understand the mental health and emotional needs of young children, in this case, young children providing care, is therefore imperative [21,36]. Organisations such as PEDAL (Play in Education Development and Learning), The Royal Foundation Centre for Early Childhood, and UNICEF-UK have launched campaigns to address the importance of infant mental health. These urge professionals and policymakers to engage in a shared understanding of what mental health looks like during early childhood [37,38,39]. Such approaches support a move away from deficit models of understanding mental health and individual needs. In the context of young carers’ needs assessment, this shift in viewpoint helps to realise that a lens of safeguarding is not enough to mitigate the impact of very young caregiving alone. Young Carer policy requires broader consideration of young carers of all ages, starting in early childhood.

### 1.2. What Can We Do?

Tyerman [40] spoke of priorities for reform within the health service, highlighting the importance of building a child health workforce, able to deliver on standards which centre children’s wellbeing at the heart of practice “*For too long and far too often, the UK’s health systems have not been required or encouraged to ‘think child*’” [40]. Knowledge of children in early childhood taking on caring responsibilities is not new to the field [41,42]. Nor was the recognition that younger carers require more targeted support [42]. However, there remains little evidence of representation in the literature of young carer discourse concerned with this foundational stage of childhood [2]. Notably lacking are the voice and representation of front-line professionals working in the arena of young carer service provision, education, and healthcare sectors, specifically concerned with the health and well-being of society’s youngest carers, YCEC. To address this gap, this study sought to understand how professionals and adults with living experience of caring in early childhood perceived the limitations of current policy and practice. The strengths and strains experienced by professionals in this context were a distinct area of focus, alongside the perceived barriers faced by YCEC and their families in accessing a needs assessment and ongoing support. Members of the Young Carers Alliance were invited to attend three focus groups, each facilitated by persons with living experience (PWLE) of caring in early childhood. Each focus group was hosted by the lead author, providing critical distance from the data collection. Each focus group presented an opportunity for professionals engaged in young carer support to convene in collective dialogue, analysed with the PTMF as the primary lens.

## 2. Materials and Methods

Ethical approval was granted by the University of Plymouth (2024-4932-6408). Recruitment of participants took place via the Carers Trust networking platforms, the Young Carers Alliance (a network involving more than 1000 individuals and 250 organisations throughout the UK, including education, health and social care, children and families services, NHS trusts, Youth services, Young carers services, Third sector organisations and academics) were invited to take part in a series of three focus groups [11]. An invitation to participate was created using Eventbrite and shared on associated social media platforms and the Young Carers Alliance newsletter. This allowed for targeted sampling of participants from across the UK. All members of the Alliance providing support for children and families, including young carers under the age of 18 (under 25 for young adult carers and young people with special educational needs, SEND), were eligible to participate.

Participants were provided with information regarding the research process to aid their decision to provide informed and ongoing consent to participate, along with debriefing information and a right to withdraw.

**Figure 1:**
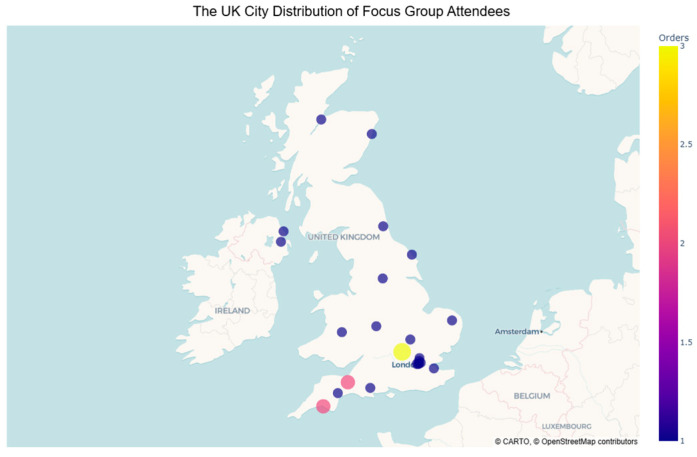
Geographical distribution of focus group attendees. Note. *Location markers are approximate to protect the anonymity of individual participants*.

Focus groups were held online using Zoom to record each session. Each group lasted 45–60 min. Recordings were transcribed and anonymised, with participants given pseudonyms to protect their identities. The focus groups were attended by a combined total of 24 participants from across each of the four nations of the UK. Eighteen participants were female, and six were male. Four participants had a living experience of caring in early childhood; fifteen participants were currently working in the field of young carer support including local authority commissioned services and third sector organisations delivering community support, education and youth and young adult services; one participant worked with a parent carer organisation; three participants were individuals with research and policy influence, one of whom was an experienced local authority children’s commissioner; one participant was a children’s author and illustrator, Figure 1.

Each focus group session was hosted by the lead author and facilitated by a group of adults with living experience of caring in their early childhoods. Facilitators were recruited through the Carers Trust (Young Carers Alliance). Facilitators were provided with briefing information and were required to give their consent and maintain confidentiality as per the participant’s information and consent agreements.

Prior to the focus groups taking place, the host met with the facilitators (*n* = 3) to agree on discussion prompts for the focus group schedule and to clarify the aim of the sessions. Each facilitator had a specific role in helping manage the session efficiently, and guidance was shared by the University of Plymouth to manage focus groups effectively.

Deductive qualitative analysis mapped extracts of transcripts against the PTMF, with each category used as an a priori framework for the process. Anonymised transcripts were mapped under headings of power, threat, meaning, and strengths [43]. The lead researcher undertook the initial mapping exercise. A second round was conducted as part of a debrief process with two focus group facilitators and one external independent policy advisor. This helped to iteratively refine themes alongside discussion and reflections. No disagreements were encountered. However, the discussion considered the difficulty at times in distinguishing coding of threat and meaning explicitly. In such cases, persons with living experience led the final decision. Final analyses were presented to the lead author’s supervision team and one external examiner for discussion as part of the lead author’s PhD progression route. A process of member checking took place to review the analysis with a group of three participants and focus group facilitators [44]. Manual methods of analysis were applied, without the use of qualitative data analysis software [45] (see Focus Group Schedule, Appendix A).

Positionality

The lead author acknowledges her position as a practitioner-researcher with experience working in both the fields of early childhood, whole-family support, and delivery of a county-wide young carers service. These experiences, alongside the living experience of caring for a family member, shape the perspective of the research produced. As such, it forces critical reflection on how such work is interpreted. Engaging with persons with living experience throughout this process is recognised as an act of shared power. This aims to mitigate the sensitised viewpoint of the lead author inherent to prolonged exposure to power and vulnerability operating within the context of working with children and families.

## 3. Results

Participants reported that some models of service delivery disempower YCEC and their families from accessing early targeted and specialist support. This creates barriers to early identification, prevention of inappropriate caring responsibility, and assessment of needs related to caregiving. Strengths were identified through the sharing of practice and offering of experience from a range of individuals. Findings demonstrate the need to review existing mandatory guidance and unify approaches to whole-family assessment and support during early childhood to prevent and minimise the risk of escalating caring responsibilities for very young children. Engagement with the ECCE sector regarding young carer advocacy is lacking in consistency throughout the UK; consequently, limited evidence-based guidance is available to support those in a position of power to be effective in the lives of very young children and families impacted by the care needs of a family member. As a result, identification and support for YCEC is variable, and dependent upon local authority delegation of resources, awareness, and training of professionals, and developmental knowledge of those already working within dedicated young carers services.

An exemplar synoptic template is included in the appendices to demonstrate how the PTMF mapping took place (see PTMF mapping template, Appendix B).

### Power

Legal power: Participants recognised the power held within legal frameworks (social, positional power) designed to support and protect young carers in early childhood. These rights are strongly advocated for within their discussions around practice, identification, assessment, and support. However, consistent failings were highlighted within systems and infrastructure. These create barriers to rights being consistently promoted in practice throughout the UK, despite a concerted collective commitment to improved implementation through initiatives such as national young carer networks.

“The previous Young Carers Alliance forum that was about education, and there were three adult, young carers who spoke very articulately about, um, how to support young carers in transition to adulthood through the education system. But one thing they all had in common they were all under eight when they started caring. Um, now, you know, we get things like the all-party parliamentary group on caring… one of the things they said was that many young carers didn’t actually get identified and supported for ten years or more. Well, guess what? If you don’t start looking to a certain age, that becomes inevitable, doesn’t it? Um, if you start looking at, for young carers when they’re younger, you’ll find more. The challenge is about how do we support; how do we resource that, Uh, but we just we can’t deal with just ignoring it.”

(Louie)

Where the young carer strategy and subsequent policy misdirect the rights of YCEC by stipulating predetermined age ranges of local authority commissioned service support, young carers are seen to be at risk of escalating caring responsibilities for longer periods of time. This is perceived to be in the absence of high-quality, consistent multi-agency awareness and support, “areas that simply deal with that by not responding to young carers under eight, um, are actually unlawfully ignoring young carers” (Jo).

Inconsistency of legal powers was further highlighted between each of the four nations of the UK, with representatives from Northern Ireland feeling disempowered to support their youngest carers without specific mention of young carers’ rights within legislation, “Northern Ireland is very far behind. Like our legislation is from 1995, and it barely mentions young carers… England, Scotland, and Wales have had wonderful new legislation within the last ten years. Um, so we don’t even have a young carer needs assessment” (Lorna). Even where legal powers are perceived to be strong, in England, for example, participants expressed that policy is not always helpful to ensuring that assessments take place at the earliest opportunity, or that support is consistent and available when needed, “there’s holes in services and infrastructure, um, that allows some of that to take place” [missed opportunity] (Kim). Such holes refer to a lack of capacity of services, due to funding, limited resources, time-limited interventions, or a lack of specialist knowledge.

“I think what we see the other way round is that social workers, mental health workers, want to send a child to us. They want to refer and then close the case on it, because they just want it to go somewhere else because they don’t know what to do. Um, so we see that an awful lot, actually.”

(Kim)

To address these problems, it was felt that the power held by local authority commissioners holds the key to opening the door for support to YCEC. Yet, strategies on how to best support YCEC remain wholly inconsistent, due to an existing postcode lottery and inconsistent strategy throughout the UK.

“… we are not commissioned to support young carers until they are eight (Anon location), however, just literally you can have someone living on the same street that also registered with our service but the commissioner (Anon location) just so happens to be supporting from five so we have a lot of issues where schools are like, well, it’s really unfair that we’ve got this four, this five year old, but not able to support this young carer who is significantly more impacted by it.”

(Matt)

“officially, we don’t have the, um, uh, the Commission to Assess under 8’s, social services should be doing that. Um, but in reality, they don’t… social services don’t really tend to assess an awful lot of young carers in counties where it’s not the carers and local carers service that are… commissioned to do those assessments., we try to take the paperwork out of it as much as possible for all age groups. Really it is just a reflective conversation… ‘Um, so we are, uh, the, um, commissioned service in our three counties for young carers over eight years and over, but we do get some additional funding through the council to work with, under eights.”

(Kim)

Economic power: Conversations steered toward economic power, suggestive of sentiments that questioned broader issues in society of why young carers are having to provide care and the lack of economic resources available to prevent such from impacting family life.

“I think it’s very difficult and it’s down to I think it’s down to money. …We rely too much on family supporting people with disabilities and long-term health issues. And it’s just assumed that the family will pick up the slack and where there isn’t older family members we’re expecting younger family members to pick up the slack. Um. Because we’re just not putting enough in to support those with the disabilities. It doesn’t matter what age you are, but it’s it is… I think it’s atrocious. That we’ve got young people who are getting to the state where they don’t know what they need, because almost what they’re doing day to day becomes so ingrained… But it shouldn’t really be family’s responsibility, should it? That the the help should be there. So, when we sit on boards, when we’re talking strategically to people, uh, to the third sector organizations, to local authority and to health, it’s just, it all comes down to money.”

(Nina)

Participants with a living experience of caring in their early childhoods reflected on the noted lack of resources. The impact of support they received was felt in some way to normalise their caregiving responsibility, rather than actively reducing such as in young children, and when growing into adulthood.

“… went to cinema a couple of times and we, we got respite for maybe a couple of hours to do some art. But that didn’t really change anything for us. It didn’t stop us having to be carers or looking after the, after our family. It just meant that I’m about to go out for the afternoon, and I think what was really missing for us was advocacy and people listening to us and being a voice for us and and even in professional judgements about the decisions about our family and what happened to us, we we weren’t asked. And., I remember being a young carer when I was a teenager and being asked, ah, what support do you need? And I didn’t know because I’d been looking after my family for such a long time that I didn’t know what I needed because this was just normal.”

(Rianne, PWLE)

Ideological power: These reflections illustrated an ideological power within society that prevents professionals working in related fields of health, care, and education, for example, from recognising YCEC due to a lack of awareness and preconceived judgments of what may be deemed acceptable for young children within family constructs.

“… there should have been a level of kind of professional judgement or professional assumption that parents who are both physically disabled are not going to get any better, and they’re having a child. The likelihood is, or it’s inevitable that that child and any of the children that they have will become young carers at some point, regardless of the age… It was obvious that that was going to happen. And yet, I wasn’t actually identified properly until I was 14.”

(Rianne, PWLE)

The sharing of experiences from PWLE was further compounded by the ideology witnessed amongst trainee professionals. This reflected a degree of judgment of what might be considered excessive or inappropriate caring for very young children, without information to guide such thought.

“When I invited young carers, to speak with me at awareness raising things. Um, and we did did some to some student nurses and their reaction was, well, one student nurse reacted by saying, I’m just shocked. I’ve got a five-year-old and a six-year-old. And if I thought my children had to be doing (xyz), I can’t believe children of this age are, she said. It’s just not right. And the young carer responded by saying, well, actually, I’m really grateful that I had the opportunity to give, uh, to support my sister in the caring role, um, before she died, and that that was a really important part in my life. So, I think we need to be really careful and be balanced about saying it’s completely wrong, because actually, um, a lot of young carers get an awful lot out of giving as well.”

(Chris)

Such ideological power shapes stereotypes and further disempowers children and young people who provide care, from fully recognising their own needs or obtaining support at the point at which it is needed. The consensus of the focus groups speculated an unspoken social rule, *“it just feels like the elephant in the room, something where we’re, you know, we’re sort of brushing over it almost”* (Sian). This was pertinent to the cultural framing of what might be and what might not be deemed caring responsibility, *“There were certain things that we, that were reinforced culturally for me. That made me feel like I wasn’t a young carer. I was just taking care of my family”* (Ethan, PWLE).

These social rules fuelled discussion, with trepidation, surrounding misunderstandings which further silence the voice of young carers, preventing children and families from accessing support outside of specialist young carers service provision and instead seeking this from settings or provision they already encountered in day-to-day life.

“…. I almost wonder if it’s a bit like Pandora’s Box. Like they don’t want to lift the lid because they think it’s all going to suddenly come, I don’t know, bleeding out and it’s going to be this massive caseload. But yeah, but actually it’s not that at all. Um, and trying to communicate that message is, is hard.”

(Jo)

Conversations explored such perceptions, further embedding feelings of shame and fear of families surrounding the stigmatisation of reaching out for help, and the lasting impact of such experiences.

“I think for the families there’s a real fear of becoming involved in a service like us because they are worried that that will involve social care, will involve, you know, people probing into the family and trying to change. Um, and we really try and, you know, to kind of explain to people that that’s not going to happen. That’s not what we do. We’re trying to support you and keep your family, keep things as they are, but with more support… and particularly for like our addiction carers, it’s very, very I, I can’t think of a single one that I know that has raised like self-identified, you know, gone to seek out that support because that fear of services, um, just is such a barrier to then to mention to your school, you know, it would come with more people are going to come in, they’re going to probe the family, they’re going to split up the family.”

(Pete)

“Because I think for, for me, it came from my parents and their association… we had social services involved with our family quite heavily… they had a real distrust and a kind of pushback from services… I think I think there is such a stigma with the word social services. Um, I think for any family, I mean, I’m a parent myself. I have two young children, and even though I work in the job that I do, I would be fearful of having social services be involved in my family because there is a stigma. …I think it is a really kind of ingrained.”

(Rianne, PWLE)

Threat

It was felt that power forms embedded within systems and structures of society (legal, economic, ideological) present threats to relationships, both internally within family systems and externally between family members and professionals. Such threats were suggested to create barriers to the identification of YCEC.

“a lot of people just don’t believe that young people of two, three, four can be providing care. They don’t understand what that looks like, and therefore they they kind of like, oh, that can’t be happening because they’re a child. So, it’s really hard for us to even understand what being a carer looks like at such a young age.”

(Louie)

Furthermore, a shared concern presented beliefs and associated fears of parents of young carers, stemming from ideological power within families and their immediate communities, illustrating a lack of acceptance towards the phenomenon of very young caregiving. Thus, creating bias (in action and self-fulfilled) both from within and external to the family construct. This creates distress and, at times, denial, which threatens effective, impactful intervention from services.

“I think the point on parents as well is really interesting because, you know, myself and my sister obviously our parents were, uh, disabled people. Um, but we we’ve experienced a lot of resistance and hesitance around there view of us being young carers and what that means in terms of their parenting ability. And I think sometimes because there is so this taboo around young carers, that that may be this hesitance for parents to engage with services because they feel like it’s you’re not able to do that, you’re not able to provide the care, the children are doing that instead. That’s absolutely not the case. I think there’s a lot of work across all young carer services of really like breaking down that taboo and going, it’s not an attack on ability. It’s not an attack on, you know, the environment of the home. It is just a it’s a benefit to young people who are providing care.”

(Rianne, PWLE)

Threats to identification: Thoughts were expressed around variations in systemic data collection for identifying young carers across each of the four nations of the UK. It was felt that parental acceptance of children in a caring role directly influences data outputs, and these were dependent on the accessibility of such data collection systems to increase recognition consistently throughout the UK.

“I know in the Scottish census; the youngest young carers actually been identified from like three years old. And that is of course down to the parents to actually acknowledge it.”

(Thea, PWLE)

Participants concurred that accessibility of data informs the way in which resources are allocated. Where services are only commissioned to provide targeted support to children over the age of eight, YCEC are at heightened risk of vulnerability. “*That’s just the way we have always worked 8–18, which is unfortunate because there’s um young carers missing out*” (Lorna).

Where identification does take place, the support offered was not always seen as beneficial for young carers in terms of addressing their caring responsibilities and as a consequence, threatening their health and wellbeing.

“actually, identification for us didn’t really change anything because the support that was there at that time. It was a long time ago. It didn’t really scratch the surface for us.”

(Mia, PWLE)

“Um, so I find I find it very difficult, and people ask me what support the young carers need and what’s available because what I needed was advocacy. I need someone to tell me to take me to the dentist. Like I need someone to make sure I knew what a healthy meal looks like or how to wash myself. And I got that through other family members, but not. Not enough that. Yeah, not enough to advocate for my needs.”

(Rianne, PWLE)

Where support could not address the needs of the cared for, participants with living experience continued to undertake caring responsibilities that they felt were inappropriate or excessive, despite intervention.

“Young carers, yes, can be very empowered by what they do, and they get a lot from being a carer and I know I certainly did, but there are definitely things where I look back on that. I shouldn’t have been doing that as a child, and I would never want another child to do that.”

(Rianne, PWLE)

“I was doing things as a young child, as a young person I should never have been doing, and I would want no child of mine doing, you know… it wasn’t safe, and it wasn’t okay”

(Sira, Facilitator, PWLE)

Threat to safety and well-being: These threats highlighted difficulties for service providers to safeguard very young children regarding broader concerns about the assessment needs for the whole family; needs were noted as not always presenting initially as caring responsibilities.

“what we have noted is there’s a massive gap now for us for under sevens. Um, and lots of parents coming forward asking for information, um, dealing with things. And, and the initial query might not be care, the initial query might be they’re not getting along, there’s some behaviour that challenges, some concerns. You know, how to juggle the needs of both of my children. I’ve got a toddler, but you know, how’s that working? Um, but you can bet your bottom dollar there’s some sort of care involved.”

(Julie)

Economic threat: A consensus was reached across the focus groups that the lack of early identification for YCEC, and young carers generally, stemmed from economic threat, that of time and resource allocated to specialised services, economic threat to young carers directly where caring responsibilities escalate to impact safety, wellbeing, education, and opportunities. Where resources and capacity for support are limited, further strain is placed on families and young carers, portraying a perpetual cycle of injustice.

“we’ve worked with siblings who are doing some really heavy and caring… changing tracking tubes before school, trying to resuscitate a brother or sister. Um, so some really serious intervention stuff and like you say, for, for other people, you need some clinical training, uh, for some of that. But the level of responsibility and safeguarding around some of that is, is a is a major concern. Um, and I think it’s hidden I think that, um, and I think the elephant in the room for me is that actually I don’t think you should be doing some of that stuff, you know, that that we should have better services. Um, young people shouldn’t be responsible for that, that level of intervention. And I think that’s a whole topic around young carers that perhaps we don’t delve into very often… we should be, uh, doing better justice, I think…”

(Pete)

The cycle of injustice further threatened the identity of young carers. Where participants with living experience were born into families where vulnerability of developing a caring role in very early life was greater, their identity was shaped through exposure. Where identification of caring roles was delayed, society failed to understand the individual fully, and masked identities disempowered children’s agency and sense of belonging.

“(assessment)… it was the first time anyone had ever said to me, oh, yeah, you know, you are a carer. And, um, I remember that being absolutely huge. And I remember crying afterwards for hours and thinking, oh my goodness, it’s all so real. Now, I can’t believe that someone has, um, both validated kind of everything that I was thinking and feeling… they were the first ever people to ever say to me….you know, you’re not just the carer … you can step back or there are options. And, um, well, personally, for me, once that was kind of said to me, it was like, oh my goodness, I think I might have an identity other than being a carer. …it kind of became part of my, my kind of personality. Um, when actually really I just wanted to be a child or I wanted to be, you know, a young adult or just a person first.”

(Mia, PWLE)

Meaning

Disempowerment: Language used during each focus group gave reference to distrust, shame, controversy, disbelief, lack of information, and lack of agency, each encapsulating the systemic disempowerment of YCEC within society. In an attempt to create meaning, participants alluded to a lack of understanding, which hinders early identification and support of YCEC consistently both in policy and practice. Becoming comfortable with the ‘uncomfortable’ created meaning in order to challenge existing practice and explore new ways of examining practice.

“I think there’s a level of controversy around it as well. Um, young carers in general, there’s still controversy around, um, but once you enter the realm of little children, I think people feel uncomfortable with that. Um, but it’s happening, so we need to do something about that.”

(Jo)

Part of such an examination requires society to hold a mirror to its flaws and consider what might be considered inappropriate or excessive caring activity for YCEC, and why?

‘‘What what is okay for, for individuals varies hugely. Um, uh, and it’s really hard because there may be no alternative. And we saw that through the pandemic that when external services weren’t available. Yes. Lots more young people became young carers, uh, and were having to do stuff because there was no other option. Um, so we allowed it in those sort of circumstances.”

(Julie)

It was also agreed that practice with YCEC requires a unique and sensitive approach to whole family support. The presentation of young carers was understood to differ from what is most commonly associated with young carers more generally, and YCEC and their families would express their needs in a variety of ways to make meaning of their experiences. In some cases, not at all.

“quite often what’s being presented to us isn’t about uh, uh, a young child being a young carer. It’s, it’s another issue, but it’s more likely part of a range of issues that are going on for that family. And I think also that parents are not going to want to say to us that, you know, my four-year-old’s a carer because I think this again, that concern around services and interventions and then what might happen. Um, so I think that’s a worry.”

(Pete)

Where services are being provided for YCEC with targeted funding, service providers are not supported optimally to help children create meaning from their experiences. Lack of resources, such as capacity to increase adult-to-child ratios, access to specialist developmental knowledge and training, or reference to clear practice guidance associated with regulatory bodies, threatens the delivery of support and intervention and compromises the intended purpose and outcomes of service delivery.

“we do work with under 8’s, Um, it’s, um, a project that I’ve sort of inherited, um and I do feel a little bit uncomfortable and a little bit anxious about it, if I’m honest. Um, just about the, the parameters of the skills that we might be needing and things like that.Um, just the legality of working with young children.”

(Sara)

Strengths

The participants enacted their agency to share experiences of strong practice and collaborated through meaningful engagement to discuss strategies that helped them provide, in some way, access to power and resources for young children and families where possible. A shared optimism countered the challenges presented with a message of hope.

“…just support all children with a kind of holistic sense of well-being, then we wouldn’t even have to be worried about identifying young carers early… so I just, I just think if we could just see each child in a more holistic way, that would be, uh, a lovely way forward.”

(Sara)

A lens of holism anchored examples of effective practice, including creating spaces of support and belonging through established local young carer communities, building relationships with children and families, and providing access to information and resources. Importantly, to help families and YCEC create meaningful change, professionals and those with living experience of young caregiving valued time and building relationships to help build an understanding of need.

“(Historically) assessments would be done very early on in the relationship with the client. But now that wouldn’t be the case. So now I would really work hard to establish a relationship with the client before I went to do that assessment. Um, because it does look at every area of their life. It looks at the home, it looks at, um, you know, their mental health, it looks at their family relationships. And I would never want to ask or probe these questions without knowing somebody. I think I wouldn’t probably get a real, true, accurate picture either. Um, so that’s really, really important to kind of establish that relationship first.”

(Gemma)

Access to the arts and connections to nature were valued in eliciting experiences from young children. This helped inform the creation of resources to support young children with opportunities for engagement, enabling professionals to identify their needs, for example, through storytelling and play.

“I’ve been doing an illustration project for young carers. Um, we’re kind of been supported (Anon service) and the Young Carers Alliance, and we just made a, uh, illustrated content to help professionals communicate with really young young carers. And out of these illustrations we made a free resource, which is a picture book aimed at 4 to 6-year-olds… I was trying to put myself into the place of the children and talking with them through drawings and through illustrations aimed at them… it was just about, I guess, having the time and the patience to sit next to them and to to talk to them...”

(Grace)

Advocacy is a resource that young carer services draw upon to extend their work, often beyond their remit, to raise awareness of a broad spectrum of agencies, including targeting those most likely to encounter the needs of the cared for, outside of tailored young carer services.

“I also work with the primary care networks as a kind of secondary role to help them better support carers. Um, and up until recently before this meeting, the young carers assessments wasn’t linking into the primary care networks. So, GP’s weren’t aware that their patient was a carer or that the patient was an adult with care needs. Um, so it has been really, really useful to to review it because I think it was a kind of long-established assessment that hadn’t been touched for a really long time. Um, so to give it kind of fresh eyes and, yeah, to make a lot of changes, I think is a good thing.”

(Pete)

Advocacy and relationship building were considered key components of conducting effective needs assessments with YCEC and their families, ensuring a holistic representation is created, which centres safeguarding with paramount importance.

“I think potentially for us is trying to find out things, safeguarding concerns that might come out of a conversation...you began gathering information probably from, from the, from the two main parties involved. So from the parent’s view or the Guardian’s view and then the professionals view and map in that together… you just have to use your professional judgement of… any worries or concerns… what the barriers are in place, what they’re missing out on and looking to try and alleviate that”

(Pete)

“(Assessment) it’s very young carer focused because we use outcome star or young carer star, Um, um, we’re very focused on, well, A. first of all, building a relationship, um, over a good few sessions and then really, really looking at the impact of what is going on at home. Um, whether that be educational, social, health, you know, do you need to go to the dentist, you know, really, really look into the impact and really, really home in on being young carer focused.”

(Isha)

The strengths outlined were validated by participants with living experience of caring in early childhood, where opportunities to explore positive and negative aspects of one’s experiences with a trusted adult were perceived as being important, even when this had not been available to them. This was poignant when professionals were considering the nature and purpose of support available and reflecting on future policy and practice decisions concerning YCEC.

“Although it was really difficult, we wouldn’t change it. We wouldn’t change how much responsibility we had because it’s made us who we are today.”

(Rianne, PWLE)

Discussion

The application of the PTMF lens proved to be useful in developing insight into the strains and strengths of service delivery concerned with YCEC. The lens foregrounds structural and ideological power operating within society, which may be missed by more commonly applied individual-level frameworks of assessment and analysis. For example, findings highlight a strength of ideological, legal, and economic power that feeds existing societal beliefs concerning YCEC. This informs constructs of perceived social norms of who young carers are most likely to be, and where they may be found. Such powers present threats to YCEC, impacting the availability of support to them and their families, and access to specialist services. As such, it inhibits the ability to counter the impact of caring responsibility for very young children consistently throughout the UK. This is perhaps highlighted further by the geographical spread of participants through regionally unbiased recruitment via a national organisation.

The focus groups provided space for professionals to share and reflect on practice, helping to create meaning in a climate where resources are scarce, and time is perceived as limited in the context of an overburdened health and social care system [46]. WHO [47] (p.xv) defines the public health workforce as all those participating in the implementation of ‘*essential public health functions.*’ Each of the 12 functions (public health surveillance and monitoring, public health emergency management, public health stewardship, multisectoral planning, financing and management for public health, health protection, disease prevention and early detection, health promotion, community engagement and social participation, public health workforce development, health service quality and equity, public health research, evaluation and knowledge, and access to and utilisation of health products, supplies, equipment and technologies) [47] (p. 11) play a role in the integration of a range of provisions constructed of interrelated systems concerned with three tenants; ‘core public health personnel’, ‘health and care workers’ and ‘occupations allied to health’. In the context of this research, the young carers’ sector and the ECCE sectors are fundamental operators within each of these categories. Yet, the absence of ECCE sector representation within this study, and young carer discourse more broadly, is acutely apparent [48,49,50]. This was further emphasised through the expressions of participants’ uncertainty and anxiety about how best to provide support to this age group, compounded by the absence of young carers’ rights representation embedded into sector policy, mandates, and practice guidance [51,52,53,54,55].

Lines et al. [56] note the invariability of conceptual and pedagogical frameworks that provide a foundation for interprofessional practice in the fields of health and allied health professionals concerning child protection. Children’s social care [57] highlights concern that reduced early intervention correlates with rising demand for social care support, as such resources are directed towards crisis management rather than preventative strategies and interventions. This places pressure on young carer services, particularly when local authorities set age limitations for a remit of support. Where a young carer may not meet a threshold for intervention due to age, and is not recognised as a child in need, the gap from the point at which caring begins and identification remains significant. The PTMF lens in this context offers researchers a broader viewpoint from which to explore risk and vulnerability. The framework creates space for considering both the impact of caring responsibility and subsequent threats to mental health. The value of this approach became evident through the contributions made by persons with living experience of caring during their early childhoods. Importantly, underpinning the necessity to improve early intervention and assessment of need at the earliest opportunity, and beyond diagnostic or purely developmental modes of needs assessment in isolation.

In England alone, there are currently 54,700 registered childcare providers providing places for more than 1.6 million children in their early years (0–5 years) [58]. The ECCE sector provides a pivotal opportunity for collaborative multiagency assessment of need. During this period, children and families will encounter pre- and postnatal health and care services, developmental assessments such as the 2-year progress check [59], and the two- to two-and-a-half-year health and development review undertaken by health visitors and community nurses [60]. These assessment examples generate a wealth of rich contextual information that may be pertinent to appropriate and proportionate assessment of caring need, particularly when assessing vulnerability as part of embedded in-person holistic reviews of child and family health [60]. In the absence of a single recommended tool for conducting a young carers’ needs assessment specifically in early childhood, and limited evidence base to inform an assessment of need for YCEC, using existing opportunities to prevent, reduce, or mitigate caring responsibility within a child’s early years is of paramount importance. The PTMF narrative discussion tool and general patterns hold the potential to capture a broader range of understanding for both the child and wider family needs. Drawing on the skills of the sector would also support collaborative working practices and knowledge sharing, helping to inform holistic assessments of need and better support place-based interventions.

Barriers to change were presented within idiomatic expressions often used to describe the complex and sensitive topic of very young caregiving. These expressions alluded to the perceived moral acceptability of such, suggesting it to be a *Taboo* subject which triggers shame or distress within families and communities. Others likened any suggestion of addressing the subject at the policy level to opening Pandora’s Box, viewing the area of focus as a catalyst for complications requiring actions and consequences that simply cannot be resourced sustainably. These sentiments further conceded that our youngest young carers are all too often overlooked, and to shift the focus would be controversial.

Fractured policy concerning young carers’ rights disempowers YCEC and their families, along with that of professionals in a position to help. Those providing young carers’ services on the frontline, and adults with living experience of caring in their early childhoods, reflected upon the silent tensions that exist within society, and consider that YCEC remain the ‘elephant in the room.’ This was further problematized by perceived limitations of existing assessment processes, which may restrict holistic understanding and relational practice due to the closed-questioning nature of bio-psycho-social tools, and the datafication agendas of local authorities [2].

In England, taken together, the Care Act (2014) and the Children and Families Act (2014) [5,61] work collectively to inform understanding of inappropriate and excessive acts of care. However, acts that define caring and the impact of such were felt to be subjective, dependent on individual and cultural influence, and not always indicative of a young carer’s specific needs. Gowen et al. [9] (p. 132) propose that “*care by under-5-year-olds may be deemed wholly inappropriate*”, yet the absence of data and research informed by the lived experience of YCEC misdirects what may be considered inappropriate or excessive for very young children. Whilst safeguarding children is in no doubt recognised with paramount importance, for young carers, multiple truths exist. YCEC are children primarily, yet their caregiving role is contained within their experience of childhood, and in the context of their family construct. A duality coexists, which participants with living experience ask to be recognised and not diminished through a lens of safeguarding alone. Hamilton and Adamson [62] discussed the concept of bounded agency within the living experience of young carers, throughout the life course. Every day experiences are shaped by and therefore constrained by structural forces in one’s life (social structures, access to economic resources, relationships, and culture). Agency is consequently bound within one’s immediate experience and impacted by what came before and what may come in the future. In the context of YCEC, the findings of this study concur with Hamilton and Adamson [62], who acknowledged that for children born into caring roles, or who become young carers early in their childhoods, the point at which support is made available holds the power to impact positive future well-being more greatly. Of primary importance is the ability for professionals to understand what they see, without bias, and for children to know they have been seen and understood within the bounded circumstances of their lives.

Where participants were actively providing support to YCEC, the activities offered were described as centering on relationship building. This involved activities such as reading and drawing, which allowed opportunities to engage with YCEC as more knowledgeable individuals. This created opportunities for sustained shared thinking. This work fostered agency and participation of YCEC, helping to create a sense of belonging. Achieved through examples of storytelling, play, and access to the natural world, sustained shared thinking can be described as an iterative process, engaging a pair or group of individuals in deeply sustained conversation to solve a problem or explore a given topic [63]. Initiatives such as the Young Carers Illustration Project [64] have been developed to create resources that centre YCEC as the main protagonist of a series of children’s story books. These books have been distributed to local authority areas throughout the UK and have gained an audience internationally [65]. Creating resources where children and families can identify with characters and narratives provides professionals with opportunities to engage children and families in thoughtful and intentional interaction. This opens opportunities for conversation regarding their needs and experiences with sensitive and curious enquiry, helping to empower YCEC to create meaning of their experiences with the support of adults [66]. This work could be developed in collaboration with the ECCE to help those working on frontline young carer services to become more confident in supporting the developmental needs of YCEC. Acts of caregiving and the impact of such may present less plainly to professionals during early childhood as the nature and impact of caring responsibility is contained within the living experience of the family system [3]. In addition, young children’s signs of distress may be misunderstood by those not trained in early childhood development, as suggested by participants. Questions applied to evidence-based needs assessment tools, such as the Manual Measures of Caring Activity, require young carers to offer a graduated response to analyse their positive and negative experiences of caring [67]. For example, they are asked to rate the frequency of feelings and thoughts that may indicate risk of suicidal ideation, “because of caring, life doesn’t seem worth living” [67] (p. 12). For very young children, suicidal ideation may present through behaviour and actions rather than explicit verbal expression. It is therefore imperative that professionals supporting YCEC are able to recognise and intervene appropriately with support for YCEC and their families to manage their distress [23]. An effective intervention described by Techapoonpon et al. [68] was studied through a clinical trial to observe the effect of sustained shared thinking using books to increase parental empowerment. The positive effects were found to be lasting, and the study concluded that the simplicity of the intervention could provide replicable solutions to support children’s wellbeing, of which parental empowerment is a key contributing factor. Further research is required to develop training and awareness to help both professionals and parents feel supported. This would strengthen guidance for those working with YCEC and young children and their families generally to understand and recognise vulnerability and signs of distress. Specifically, where behaviours may indicate risk of serious psychological harm [20,69].

The experiences shared by participants identify a need to reduce stigma associated with accessing support, and through the advocacy work conducted by young carers services, better integration of cross-sector practice is encouraged. Campaigns such as No Wrong Doors for Young Carers Memorandum of Understanding [70] were devised to promote integrated working through a whole-systems, whole-family approach; yet, limited evidence-based resources are available to determine how best to provide that support for YCEC [2,71]. The early years sector remains on the periphery of young carer strategy—a challenge to this resistance is needed for systemic change to address the strengths and strains of supporting YCEC.

Limitations: A significant limitation of this study is the lack of participants from the ECCE sector. Recruitment took place through the Carers Trust, Young Carers Alliance, with more than 1000 members. Lack of ECCE sector voice and representation within the networks is evident, and a recommendation is made to extend awareness campaigns to promote cross-sector collaboration, particularly with the early years sector. Consideration has been given to the selective nature of sampling drawn from a network primarily concerned with young carers, which may influence biased discourse towards advocacy for young carers and working knowledge of system failures.

The study relies exclusively on adult perspectives and reflections of persons with lived experience, which may offer a varied interpretation of meaning-making over time.

## 4. Conclusions

Fragmented approaches to whole-family support create barriers to early identification, assessment of caring needs, support, and prevention of escalating caring responsibilities for very young children. A lack of time and limited resources impacts the strength of practice currently available to young carers more broadly. Engagement with the ECCE sector regarding young carer rights and advocacy for such is insufficient throughout the UK. Consequently, identification and support for young carers in early childhood is variable, and dependent upon local authority delegation of resources, training, and developmental knowledge of those already working within dedicated young carers services.

Safeguarding training for all professionals would benefit from specific knowledge and awareness raising of YCEC throughout the UK to improve place-based support for young carers. This would better inform professionals working in the sector to undertake an informed assessment of need and recognition of vulnerabilities and indicators of psychological distress at the earliest opportunity. Such a change would contribute to the mainstreaming of young carers in society, with the hope of mitigating the associated known risk and vulnerabilities of profound consequences, including the risk of suicide in very young children.

Current statutory guidance relating to the learning, development, and care of children in their early years does not make explicit reference to young carers in the UK; consequently, providers of young carers services are not consistently well supported to assess or support their needs and meet the legal rights afforded to young carers of all ages [31]. Specifically, in England, the Department for Education must work with Ofsted (Office for Standards in Education) to align references to young carers across all inspection frameworks consistently. This must include the remit of early years, protecting accountability of legislative rights to children of all ages, beginning in early childhood. This would better inform professionals working in the sector to undertake an informed, holistic assessment of needs, and to recognize vulnerabilities and indicators of psychological distress at the earliest opportunity.

Where children face barriers to their wellbeing and learning, young carers in early childhood and their families are entitled to assert their right to have their needs considered and assessed.

**What is the implication of the main finding?**

- Recommendation is made in England to the Department for Education (DfE) to conduct a Post Implementation Review (PIR) to review the compliance efficacy of the statutory instrument, The Young Carer’s (Needs Assessment) regulations 2015. This would be considered an impact assessment (IA) with a focus on Young Carers in Early Childhood as part of a wider assessment of compliance for all young carers. Furthermore, the DfE is to work with Ofsted to align all Education inspection remits with legal duties, as outlined in the Children and Families Act 2014, in relation to young carers.
- A national review of ways in which existing measures of early childhood development can be utilised to harness known vulnerabilities of young caregiving would be of value. This work would inform existing in-person holistic assessment and reviews of child and wider family health needs, including comparative work across regions.
- Further research is required nationally to evaluate the efficacy of multiagency safeguarding practice concerning young carers in early childhood and children vulnerable to increasing caring responsibility over time. It is essential that studies include the perspectives of YCEC in psychologically safe ways.
- Greater advocacy for YCEC is required to improve data collection and understanding of epidemiological data to better identify the number of YCEC.
- Development of a PTMF Pattern for Young Carers could assist professionals in understanding both the impact of caring responsibility and the impact on the mental health of young carers over time.

## Data Availability

The datasets presented in this article are not readily available because of an ongoing study and due to ethical considerations of the participating organisations and persons with living experience. Requests to access the restricted datasets should be directed to Carly Ellicott.

## Appendix A

Focus group guiding questions/discussion prompts—Young Carers in Early Childhood.

○ Interdisciplinary Focus Groups:
○ Introductions: Participants will be asked to introduce their role and the service they represent.
○ How do/have you identified young carers in early childhood within your service?
○ What assessment measures do you use? Are these effective?
○ What barriers do you face in providing support to young carers in early childhood?
○ What common factors do you observe in the behaviour and mental health of young carers in early childhood?
○ How does current policy and legislation support or hinder your practice and delivery?
○ Do you feel well supported by your local authority to deliver services to young carers in early childhood?
○ What support do you have in place/need to deliver support to young carers in early childhood?
○ What information would be useful to you when designing and delivering services for young carers in early childhood in the future?
○ Is there anything else you feel should be discussed/explored further?

## Appendix B

**Categories of the PTMF (and ** ** Associated Questions)**	**Example**	**Discussion-Led Analysis**
Power—What has happened? Power types: - Biological/embodied power - Coercive power - Legal power - Economic and material power - Social or cultural capital - Interpersonal power - Ideological power	“areas that simply deal with that by not responding to young carers under eight, um, are actually unlawfully ignoring young carers.”	- Professionals hold institutional power that influences the support young carers receive.
Threat—What is the affect?	“…they (parents) tried to make my younger childhood less so caring like orientated. But looking back as an adult, I was still a young carer and I was still doing a lot more tasks than what a normal child would have been doing at that age, and that definitely had an impact going on later into life in adulthood… so, I think part of the responsibility in terms of actually acknowledging our young carers is through the parents, even if it’s just Um, like small tasks.”	- Young carers often do not understand their role, families hold shame, stigma around asking for or accepting support. - Young carers often do not receive support at point of diagnosis/onset of illness or disability of their family members. - Specialist services are not consistently commissioned to offer targeted support for children under 8 and where they do they encounter difficulties due to lack of developmental knowledge, lack of resource and barriers surrounding regulatory requirements. - Professionals are not well trained to understand the developmental needs of young carers in early childhood.
Threat response	“…because there was nothing after that (assessment). It just felt like putting a label on. It normalised it all and it was like… and this shouldn’t be something that you’re having to take on.”	- Othering/normalising caregiving serves to further disempower young carers. - Isolation, withdrawal from asking for further support.
Meaning—What sense is made?	“ So, if at that point somebody had sat down, with me at maybe 4 or 5 and gone, okay, so what do you do at home? What kind of, are you a young carer, what kind of caring responsibilities are are you gonna give, would not have been able to answer that question. Because when a child is born into that situation, they don’t know any different. So how can you ask? What questions do you do you ask to kind of really decipher what their experiences at home.”	- Professionals’ understanding of caregiving may differ from the perspective of the young carer, subsequently young children’s needs in the context of caregiving remain unmet. - Lack of societal awareness inhibits meaning making for very young carers.
Strengths—What access to power resources are available?	“when you go to assessment, having a bag of toys with you… different colour pens, sitting down on the floor just using your skills.” (Pete) “I’m definitely a massive advocate of arts and of a way of engaging young people through the arts, um, and outdoors as well. I love any kind of woodland or just getting children into nature. I think it brings out the best in them.” (Kim)	- Services resourced to support YCEC draw on creative ways to engage children in creative ways.
